# Exploring the underlying mechanisms of Ashitaba in the management of non-alcoholic fatty liver disease by integrating the analysis of transcriptomics and metabolomics

**DOI:** 10.3389/fmed.2023.1247851

**Published:** 2023-10-18

**Authors:** Huan Yang, Yunshan Li, Weihong Xu, Wenjuan Liu, Ying Xie

**Affiliations:** ^1^Department of Endocrinology, The Second Affiliated Hospital of Soochow University, Suzhou, China; ^2^Department of Internal Medicine, Tongren Hospital, Shanghai Jiao Tong University School of Medicine, Shanghai, China; ^3^Changning Administration Center of Public Hospital and Community Healthcare Center, Shanghai, China; ^4^Department of Endocrinology, Seven People’s Hospital, Shanghai University of Traditional Chinese Medicine, Shanghai, China; ^5^Department of Clinical Laboratory, Tongren Hospital, Shanghai Jiao Tong University School of Medicine, Shanghai, China

**Keywords:** Ashitaba, NAFLD, transcriptomic, metabolomic, FXR

## Abstract

Ashitaba seems to improve glucose intolerance and decrease triglyceride (TG) and total cholesterol (TC), which contribute to the development of non-alcoholic fatty liver disease (NAFLD). However, it remains to be explored the mechanism of Ashitaba in managing NAFLD. We determined the impact of Ashitaba on NAFLD, particularly its underlying mechanisms at the bioinformatic level. The established NAFLD mouse model was treated with or without Ashitaba, and the underlying mechanism was explored using transcriptomics paired with metabolomics. Ashitaba reduced obesity and liver steatosis in NAFLD mice. It identified 429 differentially expressed genes (DEGs) and verified 45 differential metabolites, especially those that alleviate NAFLD via the FXR signaling pathway. Our data may provide insight into the therapeutic impact of Ashitaba in the management of NAFLD and may be useful in clinical interventions for NAFLD.

## 1. Introduction

The prevalence of obesity is closely related to unhealthy lifestyles and diets, which significantly increase the risk of metabolic diseases. The estimated global prevalence of NAFLD among adults is 32% (1). Non-alcoholic fatty liver disease (NAFLD) has emerged as a critical public health concern in recent years, adding to the burden of chronic liver diseases (2). NAFLD is characterized by the accumulation of excess lipids in hepatocytes, leading to non-alcoholic fatty liver (NAFL) or non-alcoholic steatohepatitis (NASH) and even progression to more severe conditions such as liver cirrhosis and hepatocellular carcinoma (HCC) (3–5). Despite extensive study over the decades, there are still insufficient pharmacological interventions to impede its development.

NAFLD is a prevalent liver condition with a complex pathogenesis involving multiple signaling pathways and molecules. The potential targets for treating NAFLD include lipid metabolism regulation, such as regulating fatty acid oxidation, cholesterol metabolism, and fat synthesis through FXR, AMPK, PPARs, and SREBPs (6). Inflammation response modulation by regulating inflammatory factors’ production and signaling transduction, such as NF-*κ*B and JNK, is another target (7). Additionally, oxidative stress regulation through Nrf2 and ROS is also a potential target for treating NAFLD (8).

Ashitaba, a traditional herb medicine in Japan and a popular health food across Asia contains numerous nutrients, including flavonoids, vitamins, and dietary fibers (9–11). The chalcones contained in Ashitaba yellow stem exudates (AEs), such as xanthoangelol (XAG) and 4-hydroxyderricin (4-HD), have been found to possess anti-obesity, anti-diabetic, anti-oxidative, anti-inflammatory, anti-bacterial, and anti-cancer properties (12–17).

Our previous research demonstrates that Ashitaba improves glucose intolerance and lowers triglyceride levels via improving insulin resistance, which is a key risk factor in NAFLD. The underlying mechanism of Ashitaba remains to be explored. Thus, we investigated the role of Ashitaba in improving NAFLD in a high-fat diet mouse model. Our data may provide some clues for clinical intervention and management of NAFLD.

## 2. Materials and methods

### 2.1. Animal models and treatments

Male C57BL/6 J mice, 8 weeks old (*n* = 16), were purchased from Nanjing GemPharmatech Co. Ltd., China, and housed *ad libitum*. A high-fat diet (HFD), comprising 60% energy from fat (Cat No. D12492 from Research Diets), was fed to all animals for 4 weeks, and then these animals were divided into two groups: HFD + vehicle (HV, *n* = 8) and HFD + Ashitaba (HA group, *n* = 8, 809/kg) groups. Our preliminary dose-ranging pre-experiments indicate that 800 mg/kg of Ashitaba can effectively improve glucose and lipid metabolism without any significant toxic side effects (data not shown). These mice were continuously fed with the HFD for another 8 weeks. Normal saline-dissolved Ashitaba dried powder, provided by Professor Zhengwu Wang, was gavaged to the animals. Mice were humanely euthanized utilizing a supra-lethal dose of sodium pentobarbital, administered at 90 mg/kg. The current animal study has been approved by the Animal Ethics Committee, Tongren Hospital, and Shanghai Jiao Tong University School of Medicine.

### 2.2. Serum biochemistry

The serum was separated via centrifugation at a temperature of 4°C, followed by immediate storage in liquid nitrogen until analysis. Triglyceride (TG) and total cholesterol (TC) were assessed using a TG and TC detection kit (A110-2-1, A111-2-1, Nanjing Jiancheng Bioengineering Institute).

### 2.3. Intraperitoneal glucose tolerance test

To conduct the intraperitoneal glucose tolerance test (IPGTT), the mice were intraperitoneally administered 2 g of dextrose/kg body weight after overnight fasting. Blood glucose levels were measured at various time intervals (0, 15, 30, 60, and 120 min) utilizing glucometers (Accu-Chek, Roche, Mannheim, Germany).

### 2.4. Liver histopathology

Liver histopathology was assessed from H&E and oil red staining slides and scanned utilizing a Pannoramic MIDI (3DHISTECH, Hungary).

### 2.5. RNA isolation and real-time PCR

RT-PCR was performed as described (18). Briefly, the total RNA from the liver was extracted using TRIzol^®^ Reagent (Invitrogen, Carlsbad, CA, United States) following the manufacturer’s instructions, followed by reverse transcription using Superscript III with random hexamer primers and 500 ng of total RNA. To amplify and detect the RNA, we used SYBR Premix Ex Taq Mixes on an ABI Prism 7,300 Sequence Detection System (Life Technologies, Foster City, CA, United States).

### 2.6. Transcriptomic analysis

Transcriptomics was conducted by extracting total RNA from liver tissue samples (*n* = 4 in each group) using TRIzol^®^ Reagent (Invitrogen, Carlsbad, CA, United States). The concentration and quality of total RNA were evaluated using the NanoDrop 2000 Spectrophotometer (Thermo Fisher Scientific, Waltham, MA, United States) and the 2100 Bioanalyzer system (Agilent Technologies, United States), respectively. A cDNA library was prepared using a TruSeqTM RNA sample preparation kit (Illumina, San Diego, CA, United States), and sequencing was performed using the Illumina NovaSeq 6000 system of CloudSeq Biotech Inc. (Shanghai, China).

The obtained clean data underwent differential gene expression analysis and functional enrichment analysis using the free online platform of Majorbio Cloud Platform.^1^ Differential gene expressions (DEGs) were analyzed with DESeq2 (19), based on |log_2_FC| >1 and *P*_adjust_ <0.05 criteria. The identified DEGs were then subjected to Kyoto Encyclopedia of Genes and Genomes (KEGG) pathway analysis and Gene Ontology (GO) enrichment analysis, with a pathway deemed significantly enriched when *P*_adjust_ <0.05.

### 2.7. Non-targeted metabolome analysis

The liver samples (*n* = 6 in each group) were immediately placed on ice to preserve their integrity. To prepare the samples for LC-MS/MS analysis, 50 mg of each liver sample was utilized for the extraction of metabolites The LC-MS system was used to acquire all samples, following machine orders. The analytical conditions were as follows: UPLC column, Waters ACQUITY UPLC HSS T3 C18 (1.8 μm, 2.1 mm × 100 mm); column temperature, 40°C; flow rate, 0.4 mL/min; injection volume, 2 μL; solvent system, water (0.1% formic acid):acetonitrile (0.1% formic acid); and gradient program, 95:5 V/V at 0 min, 10:90 V/V at 11.0 min, 10:90 V/V at 12.0 min, 95:5 V/V at 12.1 min, and 95:5 V/V at 14.0 min.

The ProteoWizard program was used to convert the original data file to mzML format, and XCMS software was used to handle peak extraction, alignment, and retention time correction. To correct the peak area, the “SVR” method was utilized, and peaks with a deletion rate greater than 50% in each sample group were filtered. The metabolic identity information was obtained by scanning the laboratory’s self-built database and combining it with the public database and MetDNA. Finally, R software was used to conduct statistical analysis, including both univariate and multivariate statistical analyses. Univariate statistical analysis employed student’s *t*-test and variance multiple analysis, while multivariate statistical analysis included partial least squares discriminant analysis (PLS-DA), orthogonal partial least squares discriminant analysis (OPLS-DA), and principal component analysis (PCA).

### 2.8. Statistical analysis

For image analysis and data visualization, we utilized specialized software such as Zen Blue for microscopy adjustments, ImageJ for quantitative image assessments, Illustrator for high-resolution figure preparation, Photoshop for image editing, and GraphPad Prism for statistical plotting. The data points presented in the graphs reflect the mean values, accompanied by the standard error of the mean (SEM). To ensure robustness and reproducibility, all experiments were conducted with a minimum of three biological replicates and involved three to eight mice per experimental group. Statistical comparisons between groups were performed using the independent-samples *t*-test for normally distributed data, and non-parametric tests were applied for data sets that did not follow a normal distribution. A significance level (alpha) of 0.05 was employed to interpret statistical differences.

## 3. Results

### 3.1. Ashitaba alleviated HFD-induced obesity and liver steatosis

**Figure 1:**
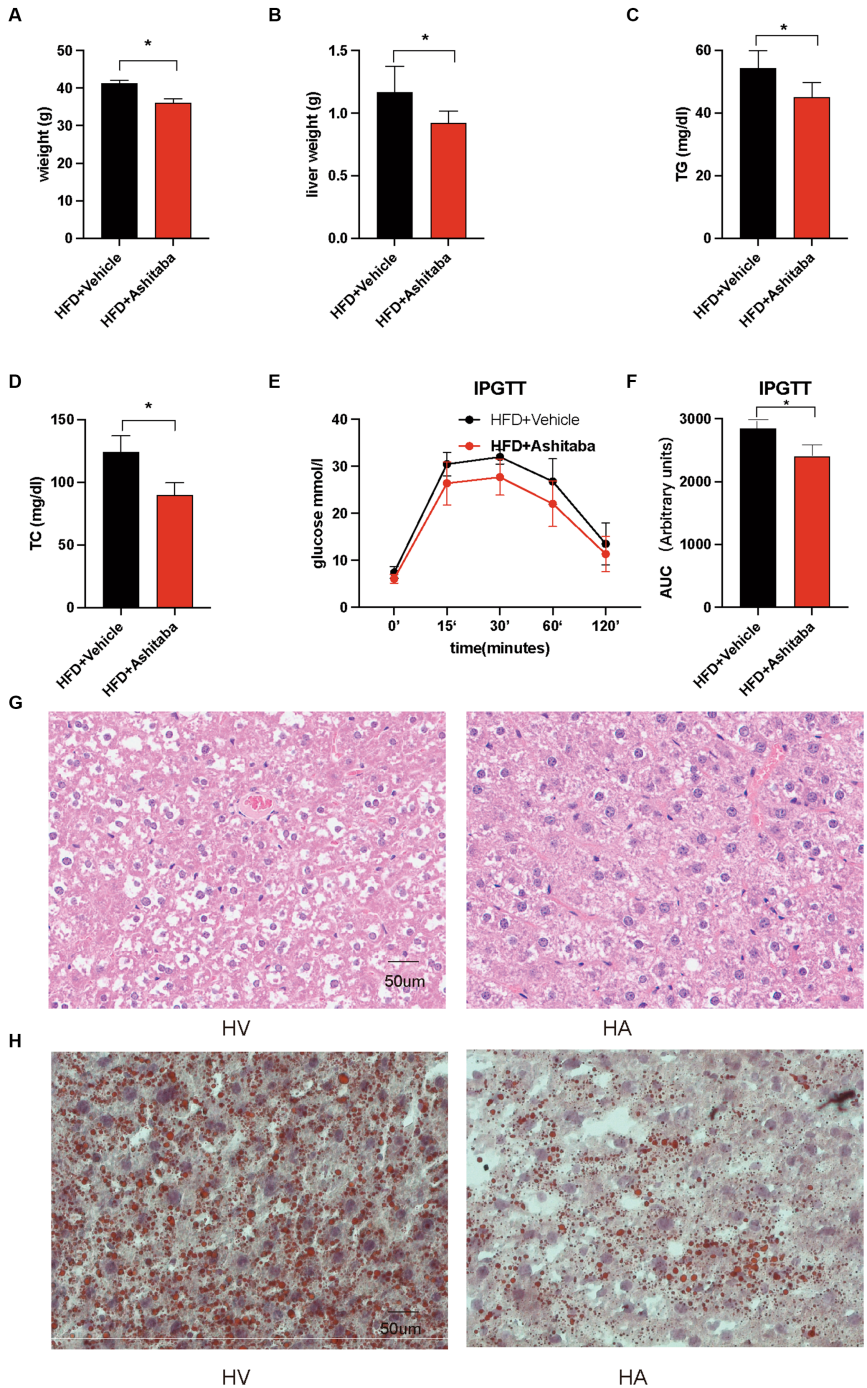
Ashitaba ameliorated HFD-induced NAFLD. **(A)** Body weight of different groups of mice (*n* = 8, ^*^p < 0.05). **(B)** Liver weight of different groups of mice (*n* = 8, ^*^p < 0.05). **(C,D)** Serum levels of TG and TC (*n* = 8, ^*^p < 0.05). **(E,F)** IPGTT of the two groups of mice (*n* = 8, ^*^p < 0.05). **(G)** H&E staining of liver sections. **(H)** Oil red staining of liver sections.

There was a significant decrease in body weight (12.6%) in the HA group following 8 weeks of Ashitaba treatment compared to the group without Ashitaba (*p* < 0.05, Figure 1A). Consistent with this, there was a reduction in liver weight in the HA group (0.92 ± 0.09 g versus 1.17 ± 0.21 g, *p* < 0.05, Figure 1B) following Ashitaba treatment. Notably, significantly reduced serum TG (82.9%) and TC (72.4%) levels were observed in the Ashitaba-treated group compared to those without Ashitaba (Figures 1C,D), which improved glucose tolerance in HFD mice treated with Ashitaba (Figures 1E,F). Additionally, we observed a significant reduction (32.9%) in liver fat droplets in the HA group using H&E staining (Figure 1G). Oil red staining also revealed a significant reduction in lipid levels within the liver of mice treated with Ashitaba (Figure 1H). The above data indicated that Ashitaba alleviated obesity and hepatic steatosis induced by a high-fat diet.

### 3.2. Transcriptomic alterations

**Figure 2:**
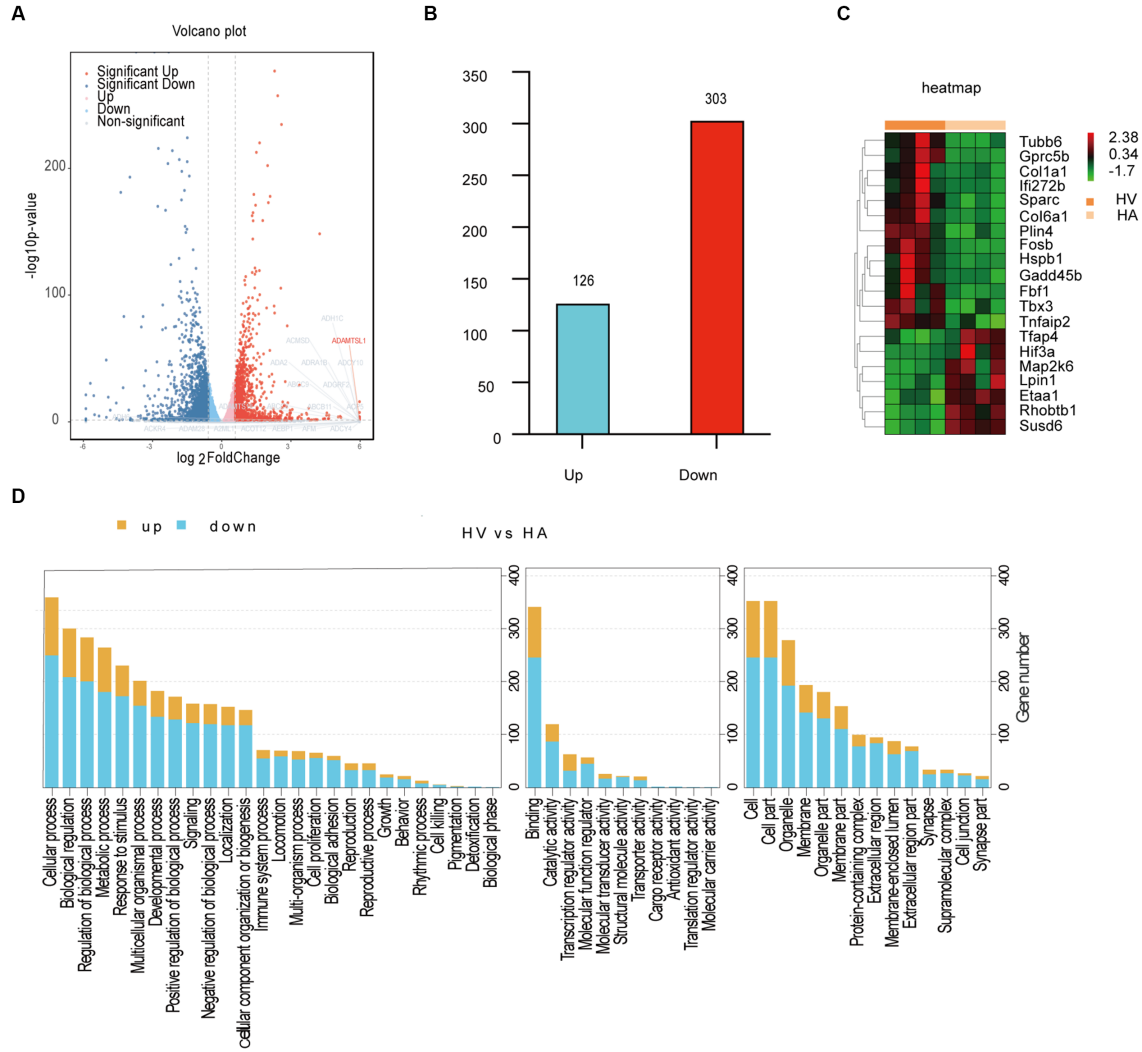
Transcriptomic analysis in mouse livers. **(A)** Volcano plots differentially expressed genes. **(B)** Upregulated and downregulated genes. **(C)** Heatmap of differentially expressed genes. **(D)** GO enrichment analysis of DEGs.

**Figure 3:**
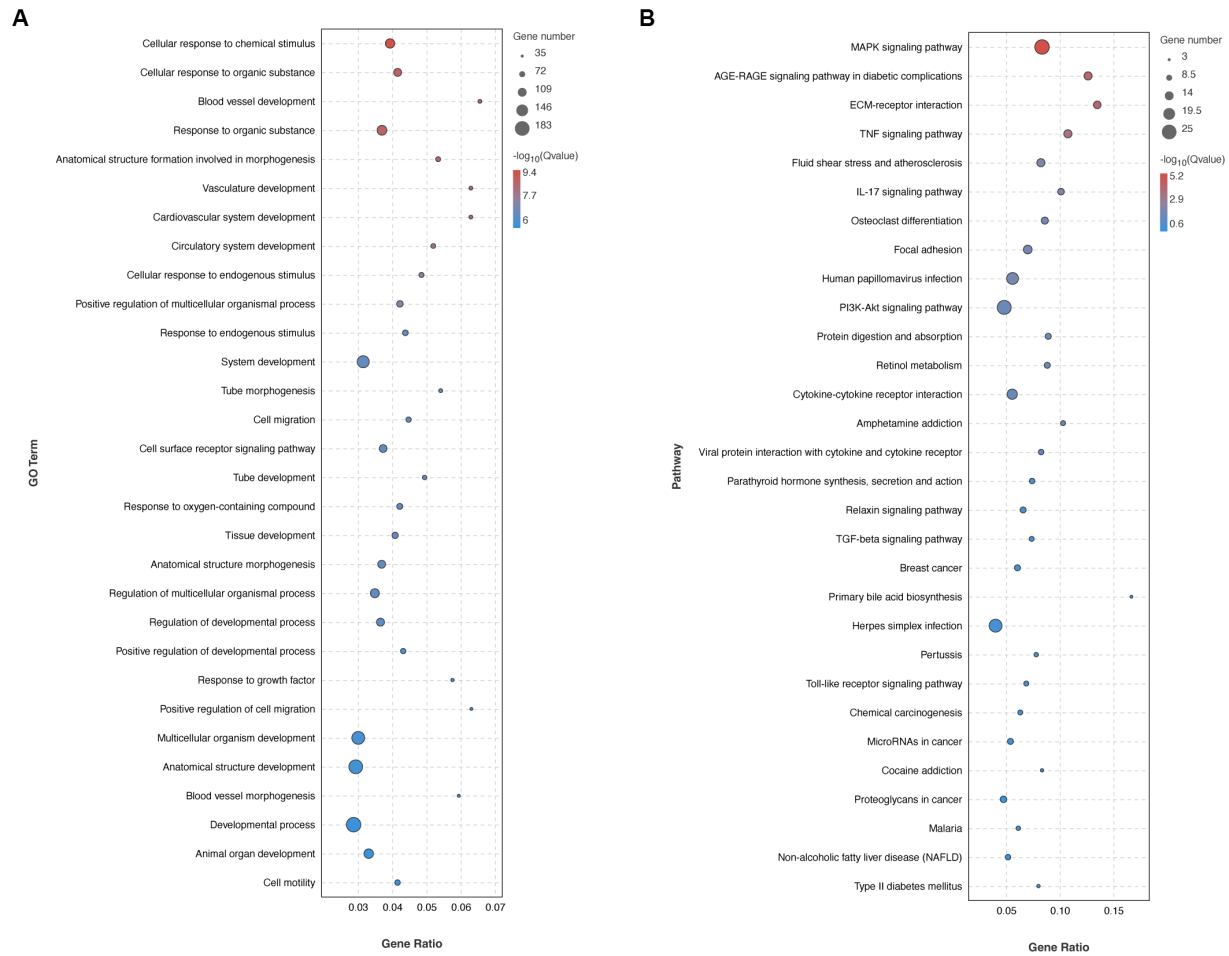
Transcriptomic analysis in mouse livers. **(A)** GO enrichment analysis of DEGs. **(B)** Pathway enrichment analysis of DEGs.

It was conducted as a comprehensive analysis by determining DGEs between HV and HA groups, utilizing RNA sequencing (RNA-seq) (Figure 2A), which was displayed with volcano plots. The liver gene expression profiles between HV and HA groups were markedly different, with a total of 429 DGEs identified (126 upregulated and 303 downregulated DEGs; Figure 2B). The log2 fold change hierarchical clustered heatmap illustrated the distinct transcriptional profiles between HV and HA (Figure 2C), using the top 20 DEGs (this number was utilized to enhance clarity). GO enrichment analysis of DEGs revealed several enriched pathways, including organic substance, developmental process, and anatomical structure development (Figure 3A). Furthermore, KEGG enrichment analysis of DEGs identified several enriched pathways, such as MAPK and AGE-RAGE signaling pathways in NAFLD and diabetic complications. In summary, the enrichment analysis of the liver transcriptome using 429 differentially expressed genes suggests that Ashitaba intervention in high-fat-fed mice may impact liver signaling pathways such as organic substances, MAPK, and AGE-RAGE.

### 3.3. Metabolomic changes

**Figure 4:**
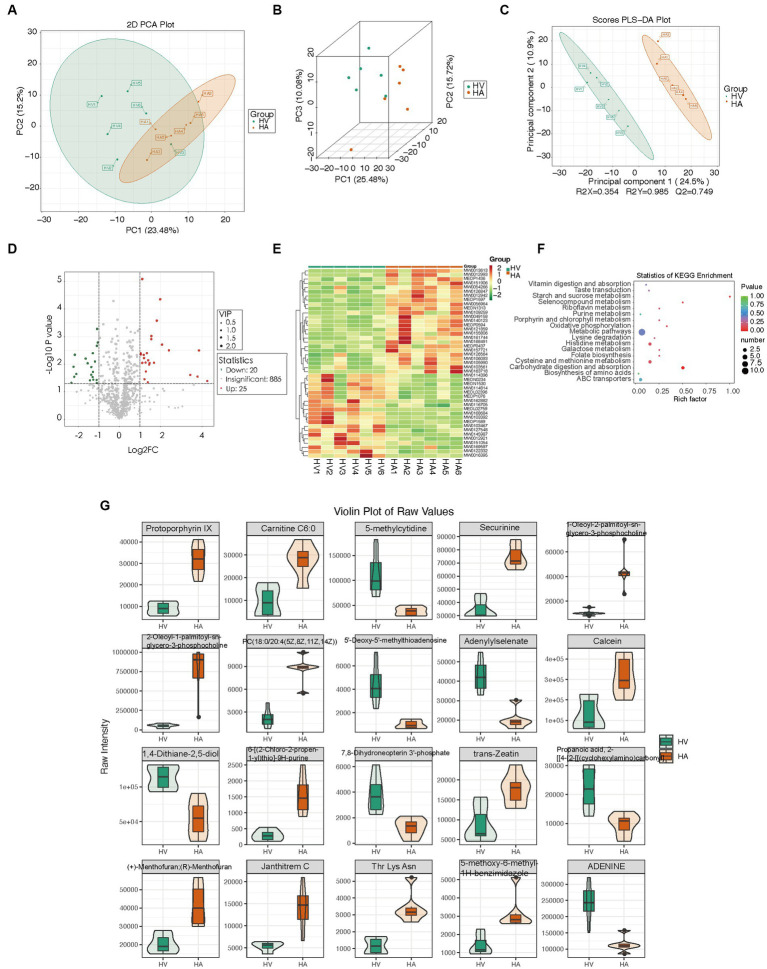
Metabolomic changes in mouse livers. **(A,B)** PCA score plot of different groups. **(C)** OPLS-DA score plot of different groups. **(D)** Volcano plots of different metabolites. **(E)** Heatmap of the relative abundance of significantly different metabolites between groups. **(F)** KEGG enrichment analysis of different metabolites between groups. **(G)** Representative differential metabolites.

To assess the discriminatory metabolites, principal component analysis (PCA) analysis was conducted, along with hierarchical clustering and correlation analyses. The quality control (QC) samples were tightly clustered within the PCA model diagram, indicating the stability of the instrument throughout the experiment. There were significant metabolic differences between the various groups (Figures 4A,B). The orthogonal partial least squares discriminant analysis (OPLS-DA) score plot demonstrated a distinct separation between the HV and HA groups (Figure 4C). Significantly upregulated metabolites were represented (red), downregulated metabolites (green), and insignificant metabolites (gray) (Figure 4D). The hierarchically clustered heatmap was presented (Figure 4E), using all 45 metabolites (25 upregulated and 20 downregulated metabolites). KEGG enrichment analysis of metabolic differences illustrated that enriched pathways encompass starch and sucrose metabolism, selenocompound metabolism, carbohydrate digestion, and absorption (Figure 4F). The 25 metabolites with the most significant differences are individually displayed using violin plots (Figure 4G). Liver non-targeted metabolomic analysis identified 45 differentially expressed metabolites, and further enrichment analysis of these metabolites suggested that Ashitaba treatment primarily influences metabolism-related signaling pathways.

### 3.4. Interactome network analysis of the transcriptome and metabolome

**Figure 5:**
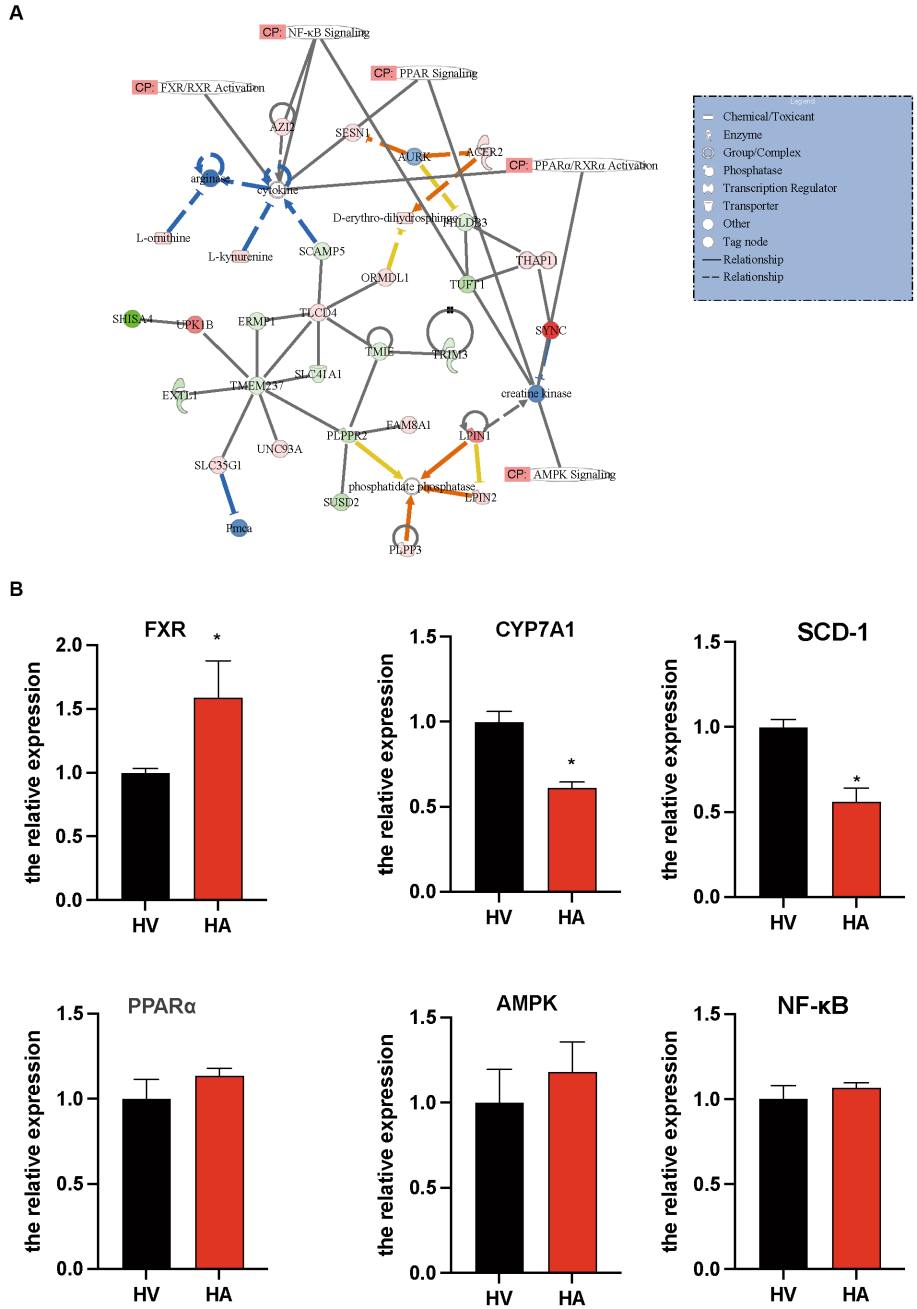
Pathway involved in the regulation of anti-NAFLD by Ashitaba. **(A)** Ingenuity pathway analysis of transcriptomic and metabolomic. **(B)** mRNA level of FXR, CYP7A1, SCD-1, PPARα, AMPK, and NF-*κ*B in the liver was examined by qPCR assay. In all panels, *n* = 3 independent experiments, data are shown in mean ± SEM, ^*^p < 0.05.

The interactome network analysis of the transcriptome and metabolome was conducted to establish the connection of pathways via gene-metabolite interactions. Following Ashitaba treatment, some key pathways were altered during the development of NAFLD, such as FXR/RXR (20), NF-*κ*B (21), AMPK (22), and PPAR (23) (Figure 5A). The interactome network illustrated that L-ornithine, L-kynurenine, and D-erythro-dihydrosphingosine contributed to the amelioration of NAFLD (Figure 5A). Thus, our data above suggest that Ashitaba ameliorates NAFLD by regulating FXR/RXR, NF-*κ*B, AMPK, and PPAR signaling pathways. To quantify the alternation of these genes regulating NAFLD with Ashitaba, we employed qPCR to analyze these pathways. It was found that the expression of FXR in the HA group increased significantly (1.58-fold), and subsequently, the expression of CYP7A1 and SCD-1 decreased by 38.8% and 43.8%, respectively (Figure 5B). However, no differences in PPAR, AMPK, or NF-*κ*B signaling pathways were noted between the two groups. Transcriptomic and metabolomic analyses of the liver, along with subsequent real-time quantitative PCR validation, suggest that Ashitaba may primarily ameliorate fatty liver through the FXR signaling pathway.

## 4. Discussion

Given the absence of effective drugs for NAFLD management, there is a pressing need to explore novel medications that can potentially halt the progression of the condition. While insulin sensitizers and various medications have been utilized in NAFLD control, their application is hindered by potential toxicity and side effects (24–26).

Therefore, the safety and toxicity of Ashitaba are also of utmost concern to us. The safety profile of Ashitaba has undergone comprehensive evaluation through a battery of rigorous good laboratory practice (GLP) tests. These tests encompassed a bacterial reverse mutation test, a chromosome aberration test, an *in vivo* mouse micronucleus test, acute oral toxicity assessments, and a 13 weeks oral toxicity study (27). In both male and female Sprague–Dawley rats, a decline in platelet counts was observed—a foreseeable outcome considering the recognized antithrombotic attributes of certain bioactive chalcones. It is noteworthy that the reduction in platelet count exhibited a marginal degree, devoid of toxicological significance in the absence of other clinical indicators (27). Notably significant elevations in serum alkaline phosphatase, total cholesterol, serum phospholipid, and triglyceride levels were discerned in rats administered the highest dosage of Ashitaba Chalcone powder (1,000 mg kg^−1^ body weight). This observation aligns with prior knowledge of Ashitaba’s impact on lipid metabolism and cholesterol transportation, thus marking an unsurprising finding.

Intriguingly, we observed that Ashitaba exhibited the potential to ameliorate NAFLD progression, potentially by modulating underlying signaling pathways and concurrently reducing triglyceride (TG) and total cholesterol (TC) levels. It is widely acknowledged that elevated TG and TC levels play pivotal roles in NAFLD development (28), aligning with our present investigation. Moreover, we noted reductions in both body weight and liver weight subsequent to Ashitaba treatment in NAFLD mice. Consequently, Ashitaba might mitigate NAFLD, in part by influencing body weight and liver weight, a finding consistent with prior evidence that these factors contribute significantly to NAFLD onset (29). Notably, glucose tolerance exhibits an inverse relationship with NAFLD development; for instance, individuals with diabetes mellitus are susceptible to NAFLD (30). Our findings indicated enhanced glucose tolerance in NAFLD mice treated with Ashitaba, providing further substantiation for Ashitaba’s potential role in NAFLD management.

Subsequently, we identified the potential regulatory genes following Ashitaba treatment. Notably, there was no significant difference observed in the liver’s lipid metabolic pathways between the HV and HA groups, as revealed by transcriptomic analysis. We attribute this to the relatively short-term (8 weeks only) nature of Ashitaba treatment, which contrasts with C57BL/6 mice that underwent long-term treatment (16 weeks) (31). In addition, transcriptomic analysis may be unable to provide clear signaling from the small number of liver samples (*n* = 4) following Ashitaba treatment, partially due to the high noise from the background genes. To overcome such a problem, we are planning to extend the sample number from each group in future, which may substantially reduce the noise/signal ratio.

In order to explore deeper into the pertinent factors of relative metabolism, we used pathway enrichment analysis; however, no conventional NAFLD-related metabolites such as spermidine (32), glutathione (33), or phosphatidylcholine (34). Under our supervision, there was the suppression of carbohydrate consumption and absorption of metabolites following Ashitaba treatment, suggesting a potential role of Ashitaba in improving NAFLD, which is consistent with reducing liver weight or losing overall body weight.

To investigate potential correlations and effects from RNA to metabolites, we integrated transcriptome and metabolome analyses. It was observed that Ashitaba may improve NAFLD through the activation of FXR/RXR, AMPK, and PPAR (23, 35–37) signaling pathways. Additionally, notable alterations in metabolites were identified, including L-ornithine, L-kynurenine, and D-erythro-dihydrosphingosine, involving the improvement of NAFLD following Ashitaba treatment. However, it remains to be clarified whether the precise underlying linkage among these metabolites and signaling pathways following Ashitaba treatment occurred during the development of NAFLD.

Our investigation further confirmed Ashitaba’s activation of hepatic FXR signaling pathways while not affecting PPAR, AMPK, or NF-*κ*B. Bile acids function as signaling molecules, orchestrating lipid metabolism, and inflammation through the nuclear farnesoid X receptor (FXR) and the Takeda G protein-coupled receptor 5 (TGR5) (38). Activation of FXR leads to CYP7A1 transcriptional repression and subsequently impairs bile acid synthesis (28). FXR also downregulates stearoyl-CoA desaturase 1 (SCD-1) to inhibit *de novo* fatty acid synthesis in the liver (39, 40). These studies lend support to our discovery of significant downregulation in CYP7A1 and SCD-1, both downstream molecules of FXR.

SCD-1 is responsible for lipid synthesis *de novo* in hepatocytes (41). We observed the upregulation of FXR and subsequent downregulation of CYP7A1 and SCD-1 in response to Ashitaba treatment in NAFLD animal models *in vivo*. This finding not only demonstrates its potential therapeutic role but also sheds light on the underlying mechanism of Ashitaba during NAFLD development. Our hypothesis gains additional support from a study by Japanese scholars, illustrating Ashitaba’s capacity to attenuate inflammatory responses in the body (15). Our data contribute a mechanistic explanation of the pharmaceutically relevant pathogenesis of NAFLD. Nonetheless, due to the intricate regulatory roles of Ashitaba, further experimentation is imperative to comprehensively elucidate its mechanisms of action within the context of NAFLD.

In conclusion, our study demonstrated that Ashitaba ameliorated HFD-induced NAFLD. The integration of hepatic transcriptomics and metabolomics suggests that Ashitaba improves NAFLD via the activation of FXR signaling pathways.

## Data availability statement

The data presented in the study are deposited in the NCBI repository, accession number is PRJNA996559.

## Ethics statement

The animal study was approved by the Animal Ethics Committee, Tongren Hospital, Shanghai Jiao Tong University School of Medicine. The study was conducted in accordance with the local legislation and institutional requirements.

## Author contributions

The experimental tasks were divided among the team members, with HY and YL managing most of the experiments. WX conducted transcriptome data analysis, while WL was responsible for some animal experiments. YX took charge of the overarching experimental design. All authors contributed to the article and approved the submitted version.

## Footnotes

^1^ www.majorbio.com
