# Supplementary material for: Exploring the underlying mechanisms of Ashitaba in the management of non-alcoholic fatty liver disease by integrating the analysis of transcriptomics and metabolomics
Source: Front Med (Lausanne). 2023 Oct 18;10:1247851. doi: 10.3389/fmed.2023.1247851 (PMC10618682; doi:10.3389/fmed.2023.1247851)
Supplement: Supplementary file 1 [file Table_1.docx]

**Table 1.** Primer sequences.

| **Gene** | **Forward Primer** | **Reverse Primer** |
| --- | --- | --- |
| FXR | GTGAGGGCTGCAAAGGTTTC | TCAGTTAACAAACATTCAGCCAA |
| CYP7A1 | TACTTCTGCGAAGGCATTTGG | AGGCATACATCCCTTCCGTG |
| SCD-1 | GTGCCGTGGGCGAGG | AGCCCAAAGCTCAGCTACTC |
| PPARα | CCGAACATTGGTGTTCGCAG | TGAACTTCAACTTGGCTCTCCT |
| AMPK | GGGAAAGTGAAGGTGGGCAA | AGATGGTGTACTGATGACCTGG |
| NF-κB | CCACAAGGGGACATGAAGCA | TCCCGGAGTTCATCTCATAGT |
|  |  |  |
|  |  |  |
